# Complete genome sequence of nitrogen-fixing strain KBS related to “*Azotobacter tropicalis”* isolated from soil in Japan

**DOI:** 10.1128/mra.00613-26

**Published:** 2026-09-14

**Authors:** Satoshi Yoshioka, Mana Adachi, Ryuichi Takase, Kohei Ogura, Wataru Hashimoto

**Affiliations:** 1 Division of Food Science and Biotechnology, Laboratory of Basic and Applied Molecular Biotechnology, Graduate School of Agriculture, Kyoto University 12918, Kyoto, Japan; Indiana University Bloomington, Bloomington, Indiana, USA

**Keywords:** “*Azotobacter tropicalis*”, genome sequence, nitrogen fixation

## Abstract

*Azotobacter* is a genus of free-living and nitrogen-fixing aerobic bacteria. “*Azotobacter tropicalis”* strain KBS was isolated from soil in Japan. The genome sequence of strain KBS comprised a single circular chromosome and two putative plasmids.

## ANNOUNCEMENT

*Azotobacter* species are free-living nitrogen-fixing bacteria with agricultural importance. *Azotobacter vinelandii* has long served as a model nitrogen-fixing bacterium (1). *Azotobacter chroococcum* is a plant growth-promoting rhizobacterium (PGPR) with significant agricultural and ecological potential due to its ability to produce phytohormones and suppress plant pathogens (2). However, genomic and physiological information on other *Azotobacter* species remains limited, highlighting the need to isolate strains with broader environmental adaptability.

Soil samples collected in Higashisumiyoshi Ward, Osaka City, Osaka Prefecture, Japan (34.6227° N, 135.5295° E) in 2020 were enriched in modified Burk’s medium supplemented with mannitol for isolation of nitrogen-fixing bacteria. After aerobic cultivation at 30°C for 3 days, colonies capable of independent growth were isolated. Genomic DNA from an isolate, strain KBS, was extracted using NucleoSpin Microbial DNA (Takara Bio) according to the manufacturer’s instructions. The 16S rRNA gene was amplified using primers 9F (5′-GAGTTTGATCCTGGCTCAG-3′) and 1510R (5′-GGTTACCTTGTTACGACTT-3′), and sequence analysis identified strain KBS as related to “*Azotobacter tropicalis”* based on 99.9% identity with strain BKK.2 (AB236162, 1457/1458 bp). No genome sequence of “*Azotobacter tropicalis”* has previously been reported. Note that, quotation marks are used as follows “*Azotobacter tropicalis*” because this bacterium is not presently listed as a validly published species name in LPSN (List of Prokaryotic names with Standing in Nomenclature).

**Fig 1:**
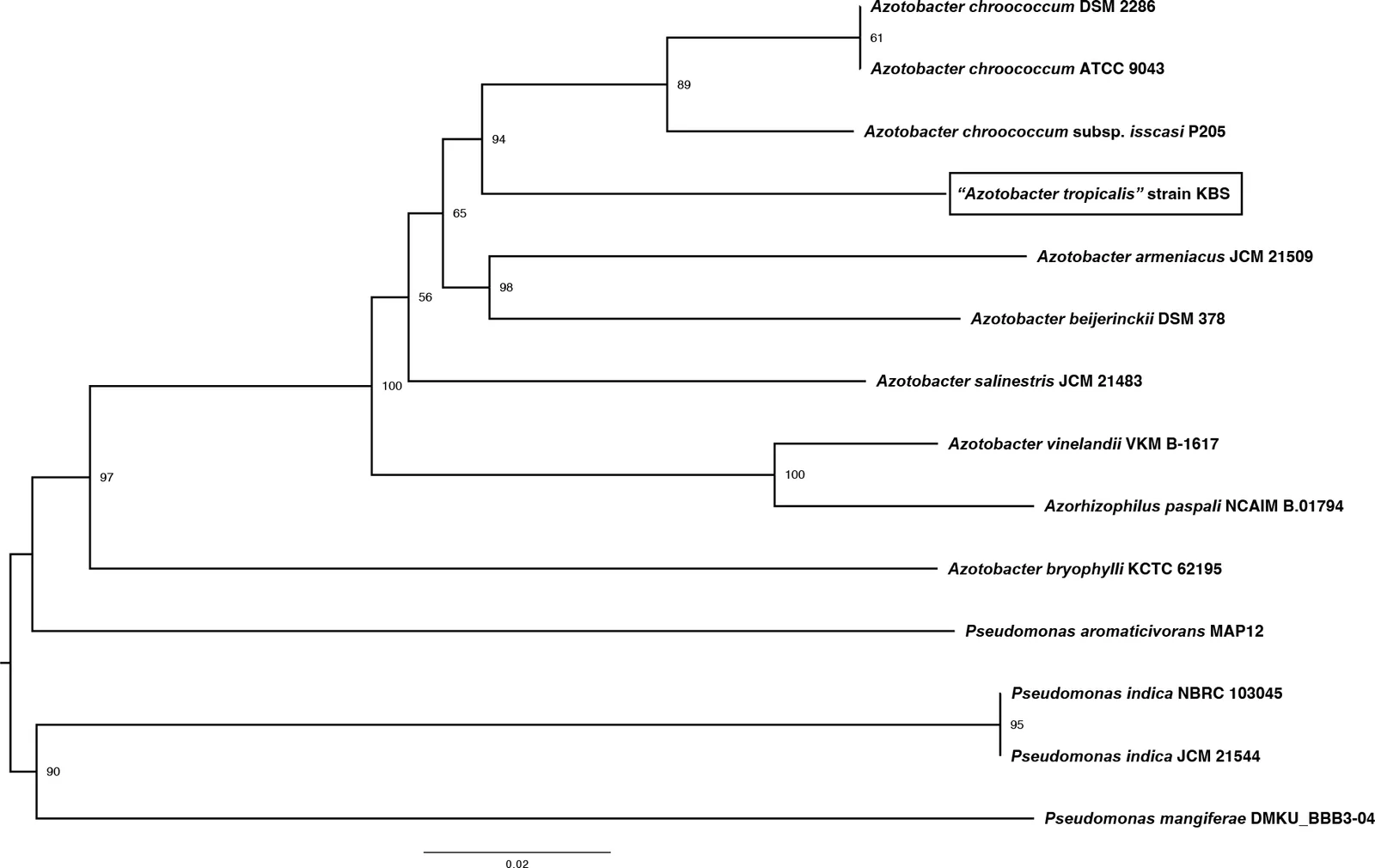
Phylogenetic tree of type strains. The tree was prepared using FastME (version 2.1.6.1) (8) based on the Genome BLAST Distance Phylogeny method implemented in the Type Strain Genome Server (9). The numbers indicate pseudobootstrap support values.

Default parameters were used for all software unless otherwise specified. Genomic DNA was sequenced using the PacBio SMRTbell Template Prep Kit on the PacBio RS II platform with P6-C4 chemistry (Pacific Biosciences), generating 143,905 reads with an N_50_ of 11,990 bp. DNA shearing or size selection was not conducted. The sequencing was outsourced to Macrogen Japan (Tokyo, Japan). *De novo* assembly was performed using Canu v2.3 (3) with the -pacbio-raw option and an estimated genome size of 5 Mb. Assembly generated three circular sequences: a 4,604,983-bp chromosome (32.43× coverage; 65.9% GC), a 360,951-bp putative plasmid (pATKBS_1; 28.30× coverage; 60.8% GC), and a 52,303-bp putative plasmid (pATKBS_2; 25.83× coverage; 64.8% GC). Total assembly size was 5,018,237 bp with an overall GC content of 65.5%. Read correction and trimming were performed internally by Canu. Self-alignment of contig ends detected no terminal overlap requiring manual trimming, and no sequence rotation was performed. The two smaller circular contigs were designated putative plasmids based on topology and annotation. Genome completeness assessed using BUSCO (v6.0.0) with gammaproteobacteria_odb10 showed 91.9% completeness (89.0% single-copy, 2.9% duplicated) (4). Genome annotation using DFAST (v1.6.0) identified 4,513,399, and 77 coding sequences in the chromosome, pATKBS_1, and pATKBS_2, respectively (5). The CRISPR/Cas system (clustered regularly interspaced short palindromic repeats and CRISPR-associated proteins) was found in putative plasmid pATKBS_1 (6). No canonical plasmid replication genes were detected by DFAST (v. 1.6.0) (7) or Bakta (Software: 1.12.0| DB: 6.0) (5). Comparative genome analysis showed that strain KBS is distinct from known *Azotobacter* species (Fig. 1), indicating a unique nitrogen-fixing lineage and providing the first genomic resource related to for future comparative and functional studies. The tree in Fig. 1 was visualized using the graphical viewer Figtree (http://tree.bio.ed.ac.uk/software/figtree/). Because no genome sequence of “*A. tropicalis*” is currently available, no reference genome could be included in the genome-based phylogenetic tree.

## Data Availability

The raw read data were registered with the DNA Data Bank of Japan (BioProject ID, PRJDB13158; BioSample ID, SAMD00553002; Run ID, Run DRR462667). The complete genome sequence of “*Azotobacter tropicalis*” strain KBS was deposited to the GenBank database under accession numbers AP046059 (chromosome), AP046060 (pATKBS_1), and AP046061 (pATKBS_2).
